# Yeast Mitochondrial Transcription Factor Mtf1 Determines the Precision of Promoter-Directed Initiation of RNA Polymerase Rpo41

**DOI:** 10.1371/journal.pone.0136879

**Published:** 2015-09-02

**Authors:** Xu Yang, Hae Ryung Chang, Y. Whitney Yin

**Affiliations:** 1 Institute for Cellular and Molecular Biology, University of Texas at Austin, Austin, TX, 78712, United States of America; 2 Department of Pharmacology and Toxicology, Sealy Center for Structural Biology, University of Texas Medical Branch, Galveston, TX, 77555, United States of America; Southern Illinois University School of Medicine, UNITED STATES

## Abstract

Despite their clear T7-bacteriophage origin, mitochondrial RNA polymerases have evolved to require transcription factors. All mitochondrial polymerases contain an extra N-terminal domain that has no counterpart in the self-proficient phage enzyme, which is therefore hypothesized to interact with transcription factors. We studied a series of N-terminal deletion mutants of yeast mitochondrial RNA polymerase, Rpo41, and have found that the N-terminal region does not abolish the effects of Mtf1; rather it contributes directly to enzyme catalysis. Mtf1 can rescue the defective Rpo41 enzymes resulted from N-terminal domain deletions. Although Rpo41 appears to have retained all promoter recognition elements found in T7 RNAP, the elements are not independently functional, and Mtf1 is necessary and sufficient for holoenzyme promoter-directed transcription activity.

## Introduction

An important function of mitochondria is supplying energy to cells via oxidative phosphorylation where the proton potential is converted to chemical energy. Genes for oxidative phosphorylation pathway components are both nuclear- and mitochondria-encoded and coordinated gene expression from both organelles is therefore essential. In addition, mitochondrial RNA polymerase can provide primers for DNA replication [1,2], thereby bestowing a critical link between transcription and DNA maintenance.

The yeast mitochondrial transcription system is a two-subunit RNA polymerase holoenzyme contains a core polymerase, Rpo41, and a transcription factor, Mtf1. Rpo41 exhibits non-specific activity because it is able to transcribe poly dA/dT templates [3], but the holoenzyme possesses promoter-specific transcription. Yeast mitochondrial promoters are quite simple, a highly conserved sequence between positions-8 to +1, relative to the transcription start site at +1, is all that appears to be required [4, 5], although the RNAP footprint extends from-15 to +15 [6]. Transcription *in vivo* invariably initiates with ATP.

Rpo41 has been described as exhibiting promoter-specific transcription on a pre-unwound duplex promoter DNA [7]. This observation implies that Rpo41 may possess an intrinsic ability to recognize a promoter sequence, but cannot do so unless the promoter is unwound, suggesting that the function of Mtf1 lies in DNA unwinding during transcription initiation. Indeed, Mtf1 was shown to dissociate from Rpo41 once the RNA transcript reaches 13 nucleotides, suggesting it is not involved in the elongation phase [8], and providing further evidence that Mtf1 participates in transcription initiation. However, Mtf1 has no demonstrable affinity for DNA [6, 9]. Structural and functional analogies have therefore been drawn between Mtf1 and eubacterial sigma factors, which also do not bind to DNA in their native, uncomplexed form. However, a crystal structure of Mtf1 unexpectedly revealed that it is nearly identical in conformation to *E*. *coli* rRNA methyltransferase ErmC' [10], and bears no resemblance to sigma factors.

Mtf1 is conserved from yeast to man, and is essential for all mitochondrial transcription. Two homologous proteins have been identified in humans, TFB1M and TFB2M. TFB1M has been shown to have methyltransferase activity and it also has been reported to function as a mitochondrial transcription factor [11, 12]. The two functions seem to be independent, as a mutant TFB1M abolishing methyltransferase activity still functions normally in transcription [13]. However, other studies do not support a transcription factor role for TFB1M [14, 15]. On the other hand, TFB2M exhibits transcription factor activity on all human mitochondrial promoters [12, 15]. TFB2M is also predicted to be a RNA methylatransferase, but this has apparently not been experimentally tested, nor has methyltransferase activity been reported for yeast Mtf1.

Although Mtf1 has no affinity for DNA, point mutations affecting Mtf1 alter promoter-dependent transcription and confer a petite phenotype *in vivo*. *In vitro*, the Mtf1 mutant Y54F is incapable of transcribing from the weak tRNA^cys^ promoter, but is active on the strong 14S rRNA promoter [16]. In contrast, the C192F mutant cannot transcribe from either tRNA^Cys^ or the variant COX2 promoter [16]. These data suggest that Mtf1 must interact with DNA in a sequence-specific manner. Using the holoenzyme, a direct interaction between Mtf1 and promoter DNA has been confirmed by cross-linking experiments [17, 18]. The finding that Mtf1 can interact with DNA in the context of holoenzyme suggests that a new DNA-binding site is formed, either by an induced-fit conformational change in Mtf1, or by the interface of Mtf1 and Rpo41.

In spite of strong circumstantial evidence for the bacterial origin of mitochondria, mitochondrial RNA polymerases show a clear bacteriophage origin. The C-terminal domain (CTD) of Rpo41 is homologous to the catalytic domain of T7 RNAP; part of the N-terminal domain (NTD) has reduced sequence similarity to T7 RNAP, but an extra ~300 amino acid domain is present that has no counterpart in the phage enzyme. Sequence similarity with the well-studied T7 RNAP offers an immediate tentative assignment of domain function, and provides a testable framework for understanding how a single component transcription system evolved into a multi-subunit system with the consequent division of functions between the enzyme and auxiliary transcription factors.

The NTD of T7 RNAP, together with the specificity loop in the CTD, is mainly involved in promoter recognition and unwinding [19]. The promoter sequence for Rpo41 (9 bp) is only half the size of the T7 RNAP promoters (17 bp), and may therefore require fewer recognition elements. The Rpo41 specificity loop has been demonstrated to interact with a promoter in an almost identical manner as that of T7 RNAP [20]. This leaves little sequence in the conserved 9 bp promoter that could bind to the extended NTD of Rpo41. The elaborate NTD may therefore perform functions beyond promoter interaction, including interacting with Mtf1 [21], and associating with a ribosome to couple transcription with translation [22]. In yeast, deletion of the N-terminal 183 residues of Rpo41 resulted in mitochondrial dysfunction without an obvious effect on transcription [23]. In humans, a naturally occurring truncated form of mitochondrial RNA polymerase, where the N-terminal 262 aa is not present due to alternative splicing, still retains RNA polymerase activity, although as a result of the loss of the mitochondrial localization sequence, the protein is transported into the nucleus [24]. These observations indicate that the NTD of Rpo41 may not participate in transcription, performing regulatory roles in other mitochondrial activities.

To investigate the function of Rpo41 NTD in promoter recognition, transcription factor interaction and catalysis, we systematically removed regions of the Rpo41 NTD and studied the properties of these variants both independently and in the holoenzyme. We report here that removal of the Rpo41 NTD does not lead to factor-independent activity, nor does it confer DNA sequence specificity, but it does reduce non-specific transcription. In addition, we show that Rpo41 displays high non-specific transcription activity in a template-conformation-dependent, but sequence-independent manner that is largely suppressed by Mtf1. Specific promoter-directed transcription activity can only be observed when Rpo41, wild-type or truncated variants, is complexed with Mtf1 in holoenzyme. Thus, in addition to its role in promoter-unwinding, Mtf1 is also required for enhancing the precision of selective transcription by suppressing the nonspecific activity of Rpo41.

## Results

### Construction and preparation of Rpo41 variants

**Fig 1:**
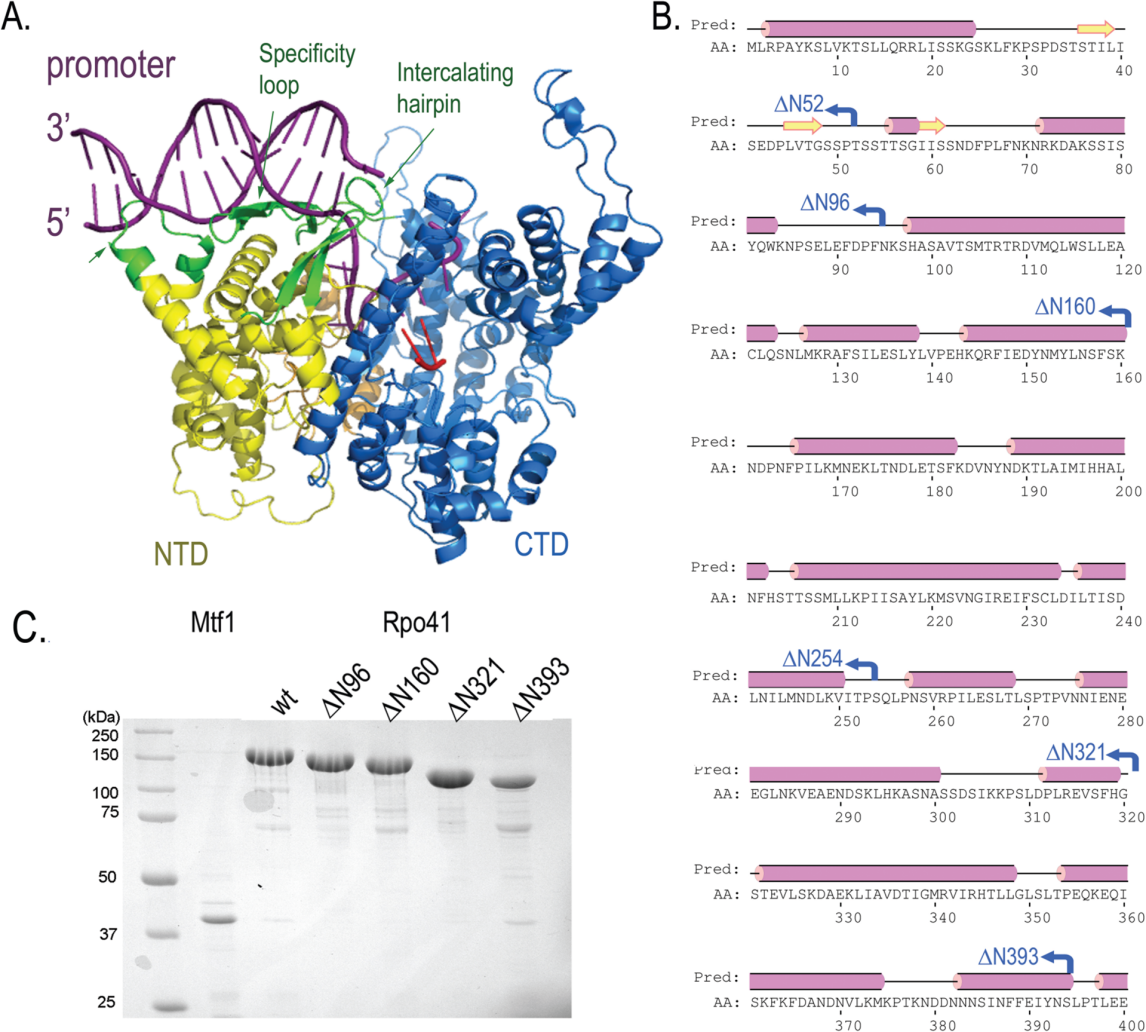
Sequence alignment and structural model. (A). a T7 RNAP initiation complex showing the 17 bp promoter (purple) is recognized by AT-rich minor groove recognition loop, the intercalating hairpin and the specificity loop (all colored green). The NTD (yellow) and CTD (blue) are indicated. (B). Primary sequence and secondary structure of the NTD of Rpo41 indicating the deletion sites. A sequence alignment of T7 RNAP with the human and yeast mitochondrial RNAPs is presented in S1 Fig (C). Purities of the Mtf1 and Rpo41 variants analyzed by SDS-PAGE.

A series of Rpo41 N-terminal deletion mutants were constructed based on its amino acid sequence alignment with T7 RNAP and predicted secondary structure. The high sequence similarity between the C-terminal domains of Rpo41 (residues P^660^-S^1351^) and T7 RNAP (P^266-^A^883^) provides an anchor point for a confident initial comparison of the corresponding N-terminal regions (Fig 1 and S1 Fig). Addition of the human mitochondrial RNAP sequence to the alignment also helped maintain the register between Rpo41 (1351 aa) and T7 RNAP (883 aa). The three promoter recognition elements of T7 RNAP (specificity loop, intercalating hairpin, and AT-rich minor groove recognition loop) are largely conserved in Rpo41, with the last two elements being located in the NTD. Compared to the NTD of T7 RNAP, the Rpo41 NTD can be divided into two subdomains, N1 (residues 1–312) that is absent in T7 RNAP, and N2 (residues 313–673) with reduced, relative to the CTD, similarity to the NTD of T7 RNAP. The AT-rich minor groove recognition and the intercalating hairpin motifs lie in subdomain N2.

Although this analysis shows that Rpo41 seems to contain counterparts to all the T7 promoter recognition elements, by itself Rpo41 has no promoter-specificity on duplex DNA. It seemed possible that the N1 subdomain could hinder any intrinsic promoter recognition activity of Rpo41. Accordingly, mutations were constructed where the N-terminal 52, 96, 160, 254, 321 or 393 residues were removed, yielding Rpo41-Δ52 (containing 1299 aa), ΔN96 (1255 aa), ΔN160 (1191 aa), ΔN254 (1097 aa), ΔN321 (1030 aa), and ΔN393 (958 aa), respectively. Rpo41 ΔN321 removes the entire N1 subdomain, whereas ΔN393 also removes the AT-rich recognition loop [25]. The deletions were chosen to be in predicted flexible regions to minimize structural disturbance and stability of the protein. All the mutant Rpo41 proteins and Mtf1 were expressed and purified to an estimated homogeneity greater than 95% (Fig 1C). Both the purification and yield of wild-type and mutant Rpo41 mutant proteins were comparable, providing confidence that the deletions had not grossly affected the overall protein structures. Preliminary activity assays on a 70 bp linear template containing a strong, 14S rRNA promoter showed that all polymerase variants exhibited some transcriptional activity, and the pairs Rpo41-ΔN52 and Rpo41-Δ96, and Rpo41-ΔN254 and Rpo41-Δ321, had similar properties (data not shown); Rpo41-ΔN52 and Rpo41-ΔN254 were therefore not further studied.

### Enzymatic activities of ΔN Rpo41 mutants

**Fig 2:**
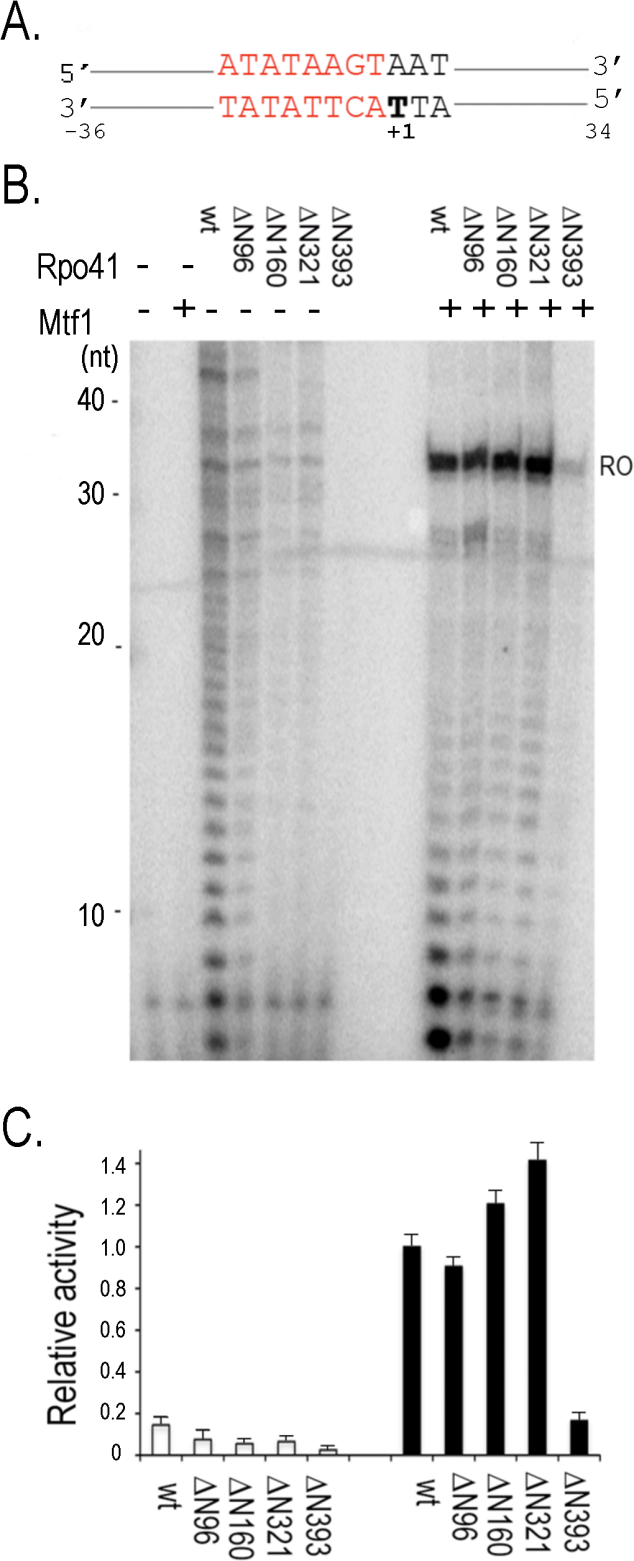
Activities of Rpo41 variants on a linear template. Transcription of 1 μM 70-bp linear DNA containing 14S rRNA (A) using 0.5 μM Rpo41 (wild-type or a variant) without or with 2.5 μM Mtf1 were performed in the presence of NTPs and [γ-^32^P]ATP (B). (C). The intensities of 34- mer transcripts in (B) were calculated by Quantity One software (BioRad).

The N-terminal deletions gradually tailor Rpo41 down to the size of T7 RNAP. If Rpo41 indeed possesses an intrinsic specific transcription activity that is hindered by the N1 subdomain, the activity would likely be revealed in one or more deletion mutants, and the role of Mtf1 would be to unlock the self-inhibition. The activities of the Rpo41 variants were determined on a 70 bp linear duplex DNA template that supports synthesis of a 34-mer run-off RNA from a 14S rRNA promoter (Fig 2A). Because correct promoter usage leads to transcription initiation with an adenosine, γ-ATP was used to visualize the products.

In the absence of Mtf1, neither the wild-type enzyme nor any of the Rpo41 deletion mutants yielded a major 34-mer RNA product (Fig 2B). Rather, a ladder of transcripts fairly evenly distributed between 7 and ~45 nucleotides was observed, indicating that these are mainly products of non-specific transcription. Using wild-type Rpo41, the band corresponding to the expected size for a 34-mer run-off product is no more prominent than several other bands corresponding to ~25 and ~42 nucleotides. Transcription activity is highest for the wild-type enzyme, and gradually decreases with progression of the N-terminal deletion from ΔN96 to ΔN321. Synthesis of short products decreases more rapidly than longer transcripts, which thereby become a higher proportion of the total transcripts made by Rpo41-ΔN160 and ΔN321. The N1 subdomain may therefore not directly contribute to the failure of wild-type Rpo41 to perform promoter-specific transcription. Rpo41-ΔN393was extremely defective in this assay, suggesting that the region of Rpo41 in subdomain N2 that is homologous to the AT-rich minor groove promoter recognition element in T7 RNAP is either non-equivalent or is not completely functional by itself. Since the non-specific products are much higher in the linear DNA than the plasmid DNA, they could arise from unannealed single stranded DNA in the assay.

Promoter specificity is completely restored when Rpo41 is combined with Mtf1 to form holoenzyme. Both wild-type or mutant Rpo41 holoenzymes synthesized predominantly the 34-mer run-off transcript (Fig 2), clearly indicating promoter-specific transcription. Thus, Mtf1 is absolutely required for promoter-specific transcription. Furthermore, with the exception of the ΔN393 holoenzyme (discussed separately below), the yields of full-length transcripts are comparable, suggesting that the N-terminal 320 amino acids of Rpo41 do not constitute a major Mtf1 binding site and that removal of this N-terminal region does not affect the ability of a holoenzyme to synthesize run-off transcripts from a linear duplex promoter. Interestingly, the mutant holoenzymes produce far fewer abortive transcripts than wild-type. Similar observations can be made with the experiments using templates with a pre-melt promoter shown below. We will return to this topic in Discussion.

Holoenzyme containing Rpo41 ΔN393 is defective, exhibiting only 20% of the activity of the wild-type enzyme (Fig 2C). The large reduction in activity, relative to ΔN321, suggests that the 72 residues between T^322^-N^393^ are critical either for Mtf1 interaction or for catalytic activity. ΔN393 is the only mutant we constructed that removes a putative homolog to the T7 RNAP AT-rich minor groove recognition motif; the defect in this mutant holoenzyme suggests that the T^322^-N^393^ region may function together with Mtf1 in transcription initiation, perhaps in opening the promoter.

**Fig 3:**
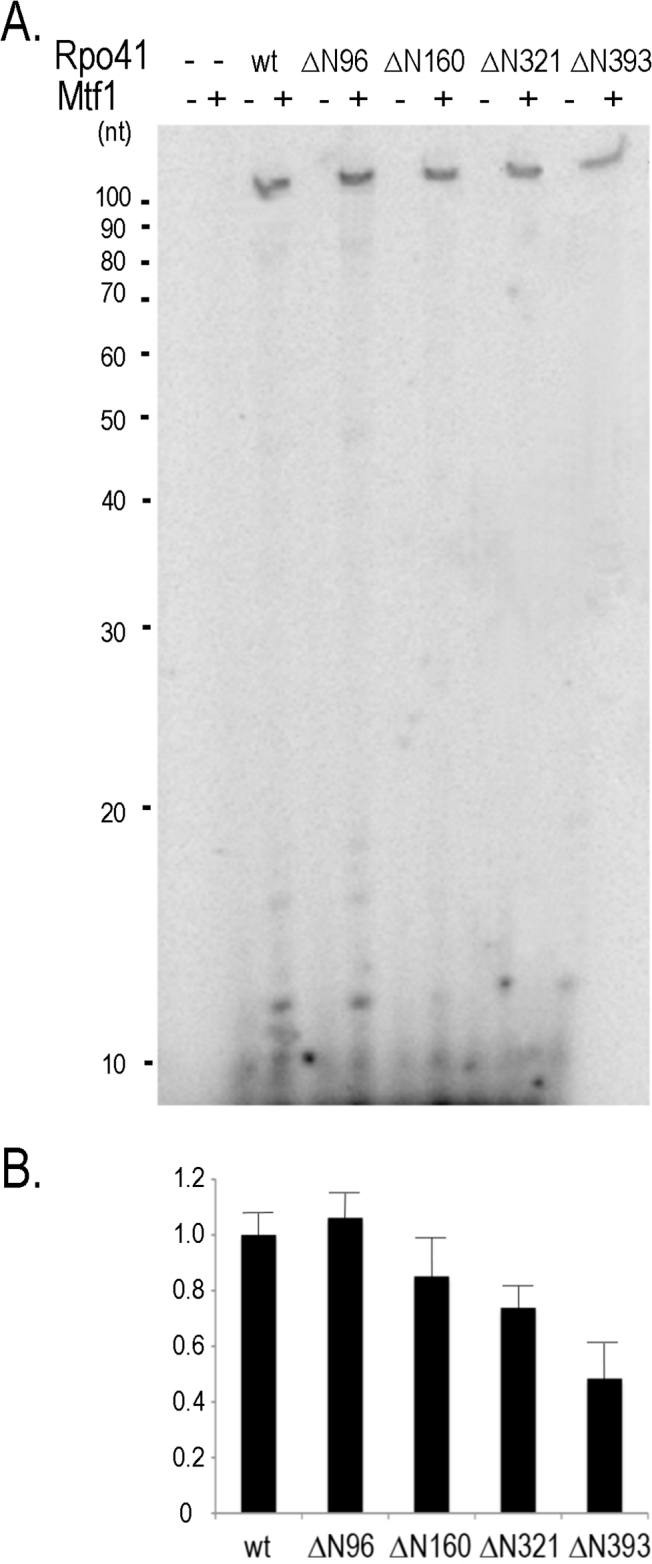
Activities of Rpo41 variants on a supercoiled DNA containing a 14S rRNA promoter. (A). Transcription was carried out in the presence of ATP, UTP, GTP and [γ-^32^P]ATP (no CTP) with 0.5 μM Rpo41 (wild-type or a variant) in the absence or presence of 0.5 μM Mtf1 using 0.2μM pJJ1305 as template. (B). The 107-mer RNA products quantified using software. Quantity One.

To test this idea, we assayed Rpo41 variants on plasmid DNA containing the 14S rRNA promoter, which supports synthesis of a 107-mer RNA if only ATP, UTP and GTP (i.e., omitting CTP) are provided. Negatively supercoiled DNA requires less energy to unwind than a linear duplex, and thus if the Rpo41 mutants are defective in DNA unwinding, they may exhibit higher activity on plasmid DNA than on a linear template. However, neither wild-type nor mutant enzyme was able to synthesize significant amounts of product in the absence of Mtf1, indicating that transcription by these mutants is not helped by a partially unwound promoter. In the presence of the transcription factor, wild-type Rpo41 and ΔN96-ΔN321 holoenzymes display comparable activities (Fig 3A), a result that is similar to the properties of the enzymes on the linear oligonucleotide template (Fig 2). In marked contrast however, Rpo41-ΔN393 exhibits significant activity on the supercoiled template in the presence of Mtf1, albeit still somewhat lower than the other variants (Fig 3B). Comparing the activities of Rpo41 ΔN321 and ΔN393 on the supercoiled and linear templates suggests that the T^322^-N^393^ region does participate (with Mtf1) in unwinding a promoter. However, as the activity of Rpo41 ΔN393 on the plasmid DNA template appears to be lower than the other mutants, the enzyme may also have a reduced affinity for either binding to the 14S rRNA promoter or to Mtf1.

### Interaction of Rpo41 variants with Mtf1

**Fig 4:**
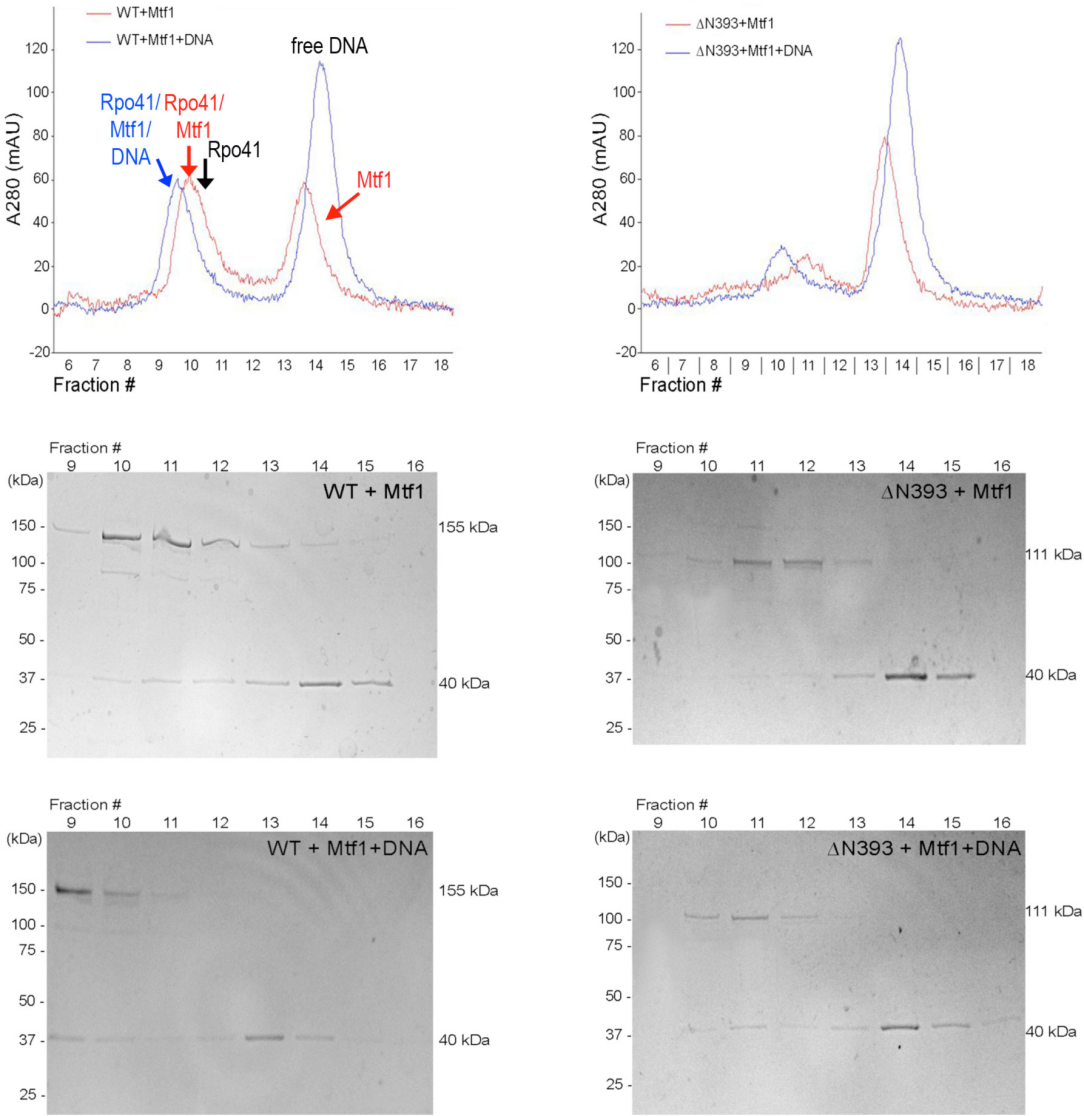
Interactions of Rpo41 with Mtf1 and promoter DNA. (A). Analytical gel filtration chromatograph of wild-type Rpo41 (2.8 μM) with Mtf1 (5.6 μM) (red) superimposed with that of Rpo41 (1 μM), Mtf1 (2 μM) and a 20bp promoter-containing DNA duplex (3 μM) (blue). The elution volume of Rpo41 alone is indicated (black arrow). The protein contents of the peak fractions of Rpo41 + Mtf1 and Rpo41 + Mtf1 + DNA were visualized after SDS-PAGE. (B). Analyses described in (A) performed with Rpo41 ΔN393.

The affinity of Rpo41 for Mtf1 was assessed using analytical gel filtration chromatography. Since Rpo41 ΔN393 was the most defective mutant, it was selected for study, together with wild-type. When Rpo41 wild-type, ΔN393 or Mtf1 are individually applied to a Superdex GL300 column, they elute as species with apparent sizes of, respectively, 150 kDa, 113 kDa and 42 kDa (not shown), in good agreement with their theoretical molecular weights 153 kDa, 111 kDa and 40 kDa. When 1 μM wild-type Rpo41 is mixed with 2 μM Mtf1, two peaks eluted (not shown), which correspond to the individual proteins and suggests that at this concentration the two proteins do not complex. Increasing the concentration of each protein 2.8-fold allows formation of a 1:1 190 kDa complex of Rpo41-Mtf1 (Fig 4A). Analysis of the peak fractions on a SDS-PAGE gel confirms that the earlier eluting peak contains Rpo41 and Mtf1 while the second peak contains only Mtf1. Densitometry scanning of the gel lanes, and making appropriate corrections for the different sizes of the proteins, suggests that ~40% of Rpo41 complexed with Mtf1. However, under the same conditions, only ~20% of Rpo41-ΔN393 interacts with Mtf1 (Fig 4B, Table 1). Taking together with results from enzymatic activity of the polymerase variants, we concluded that residues 322–393 of Rpo41 make an important contribution to Mtf1 binding.

**Table 1:** Densitometry scanning of SDS with Mtf1 and Rpo41.

Bands quantified	Rpo41+Mtf1	Rpo41+Mtf1 +DNA	Rpo41ΔN393 + Mtf1	Rpo41ΔN393 + Mtf1 + DNA
Intensity ^a^ of the upper band, I_upper_	13120	6840	5441	2580
Intensity ^a^ of the lower band, I_lower_	1311	1736	464	734
I_upper_/I_lower_	10.0	3.9	11.7	3.5
Free Rpo41^b^	8007	69	4188	598
Bound Rpo41^c^	5112	6770	1252	1981
% complex	39	99	23	77
Apparent K_d_ (μM)	4	0.02	13	0.6

^a^ Intensities were measured using fraction 10 of Fig 4A for wild-type Rpo41 and fraction

^b^ *Free_Rpo*41 = *I*_*upper*_ *—*[*I*_*lower*_ (*MW*_Rpo41_ /*MW*_Mtf 1_)]

^c^ *Bound_Rpo*41 = *I*_*upper*_ *—Free_Rpo*41

The concentrations at which complexes between Rpo41 and Mtf1 can be detected by gel filtration are however, much higher than we have used in transcription assays activities. Because RNA synthesis is dependent on the presence of DNA and both Rpo41 and Mtf1, the presence of promoter-containing DNA may enhance their interaction. We therefore repeated the gel filtration experiments in the presence of a 20 bp duplex DNA containing the 14S rRNA promoter DNA. Under these chromatographic conditions the 20 bp duplex DNA, whose actual mass is ~13kDa, elutes with an apparent size of ~30 kDa, slightly later than, but overlapping with, the 42 kDa Mtf1. When 1μM wild-type Rpo41, 2 μM Mtf1 and 3 μM DNA are mixed and then subjected to gel filtration, two peaks of apparent size 220 kDa and ~30 kDa eluted (Fig 4A). The absorption ratio at 280 nm and 260 nm indicated that both peaks contain protein and DNA. SDS-PAGE and staining for protein showed that the earlier eluting peak contains both Rpo41 and Mtf1, whereas the second peak contains only Mtf1. Quantification of the bands by densitometry indicates the molar ratio of the two proteins in the higher molecular weight peak is 1:1, and ~100% of the Rpo41 is complexed to Mtf1. The 280/260 nm UV absorption ratio of this peak is also 1:1, indicating the presence of an equimolar amount of 20 bp DNA. Thus, the 220 kDa peak represents the complete formation of a Rpo41-Mtf1-DNA complex with 1:1:1 stoichiometry. The second peak from the gel filtration column contains both Mtf1 and DNA, which almost co-elute, and is due to their being in stoichiometric excess over the amount of Rpo41. When Rpo41 ΔN393 and Mtf1 were mixed in the presence of the same 20 bp promoter DNA and chromatographed, similar results were obtained: a 1:1:1 complex was obtained that contained about 75% of the input Rpo41 ΔN393 (Fig 4B). Thus, relative to wild-type, the Rpo41 ΔN393 holoenzyme has a lower affinity for DNA. Using these data, the apparent Kd value of wild-type Rpo41 to Mtf1 can be estimated to be ~4 μM the absence of DNA but ~20 nM in the presence of DNA (Table 1). The corresponding values for Rpo41 ΔN393 are ~13 μM and ~600 nM, the latter value reflecting the combined reduction in affinity of the deletion mutant for both Mtf1 and DNA. The higher affinity of Rpo41 and Mtf1 for each other in the presence of DNA is likely a consequence that both proteins undergo induced-fit conformational changes to create a DNA-binding surface.

### Transcription on a pre-melted bubble template

**Fig 5:**
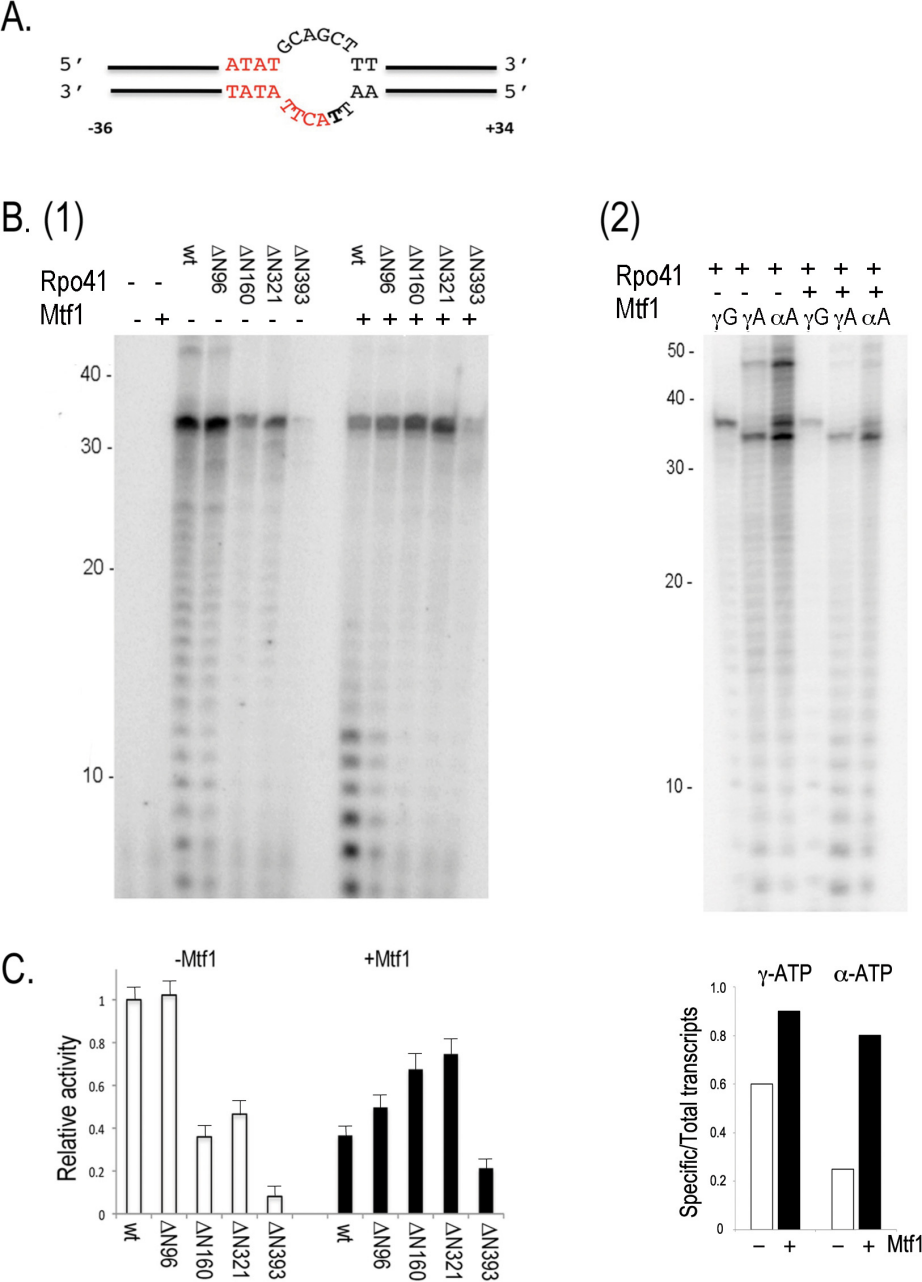
Activities of Rpo41 variants on pre-melted promoter-containing DNA. (A). The duplex DNA template contains a 14S rRNA promoter, with non-complementary sequences from positions-4 to +2. (B) (1) [γ-^32^P]ATP labeled transcripts on the above template (1 μM) using Rpo41 (0.5 μM wild-type or a variant) in the absence or presence of Mtf1 (2.5 μM) were visualized on a polyacrylamide denaturing gel. (2) Same as (1), except that transcripts are labeled with [γ-^32^P]ATP, [γ-^32^P]GTP or [α-^32^P]ATP. C) The intensities of run-off transcripts in B (1) quantified using Quantity One software.

In the absence of Mtf1, Rpo41 has been shown to transcribe from a synthetic 70 bp 14S rRNA promoter-containing DNA that has been pre-melted through non-complementary sequences spanning the-4 to +2 positions [7]. In order to examine additional properties of the Rpo41 variants, we prepared the same DNA (Fig 5A). This template allows the enzymes to bypass the promoter-unwinding step and to directly initiate transcription.

In the absence of Mtf1, wild-type Rpo41 was competent to synthesize significant amounts of a run-off product of 34 nucleotides (Fig 5B(1)). Although the ladder of non-specific transcription products seen on a duplex DNA promoter (Fig 2B) is still present, the majority of RNAs synthesized corresponds to transcription initiation at the pre-melted promoter and yield full-length products. Rpo41-ΔN96 synthesized about the same amount of 34-mer run-off product as wild-type (Fig 5B and 5C), although because ΔN96 exhibits less non-specific initiation the run-off product becomes a somewhat higher fraction of all RNAs made. Similarly, although Rpo41 ΔN160 and ΔN321 only synthesized about half the amount of run-off transcript, the latter represents the majority product. In contrast, Rpo41 ΔN393 is only able to synthesize a trace amount of any product.

### Effects of Mtf1 on mutant Rpo41 activities

In contrast to the stimulation of all Rpo41 deletion mutant activity on a linear duplex promoter DNA, on this pre-melted promoter these Rpo41 variants respond to Mtf1 differently. Wild-type and ΔN96 are both inhibited by Mtf1; the corresponding holoenzymes synthesize less than half of the amount of run-off product synthesized by the polymerases alone (Fig 5B(1) and 5C). To the contrary, Rpo41 ΔN160, ΔN321 and ΔN393 are all stimulated by Mtf1: twice the amount of run-off products are synthesized by the corresponding holoenzymes than made by the polymerases alone. Consequently, the amount of run-off products made by Rpo41 ΔN160 and ΔN321 are greater than wild-type holoenzyme (Fig 5). Interestingly, and perhaps non-coincidentally, the change of response to activation by Mtf1 occurs with Rpo41 ΔN160, the longest variant enzyme that shows reduced independent polymerase activity. Nevertheless, these data clearly demonstrate that Mtf1 compensates for the abrogated activity due to truncations of the N-terminal 321 residues of Rpo41. Mtf1 however, cannot completely rescue the deletion mutant ΔN393, providing confirmatory evidence that the 72 residues of Rpo41 between T^322^-T^393^ are involved in interacting with Mtf1.

### A pre-melted template DNA allows promoter-independent transcription

While studying the transcription activities of Rpo41 variants on the pre-melted promoter DNA, we observed unexpectedly high activity on a control, non-promoter-containing DNA. The design of this control template potentially allowed transcription from both strands and also initiation with GTP. We found that Rpo41 initiates efficiently with either GTP or ATP and we found transcripts originating from positions other than +1 (data not shown). Even more unexpectedly, the pre-melted 14S rRNA promoter also showed aberrant transcription from sites other than the +1 position. Consequently, we tested for incorporation of [γ-^32^P]ATP on the pre-melted 14S rRNA promoter.

In the absence of Mtf1, and labeling reaction products with [α-^32^P]ATP, Rpo41 makes two RNAs of about the expected size for a run-off transcript, and a less abundant transcript of ~45 nucleotides (Fig 5B(2)). The latter is also present when [γ-^32^P]ATP is used as the label, and thus the RNA is initiated with ATP but it is not obvious from the sequence of the template DNA where transcription started. Only one of the two bands close to expected run-off size was initiated with ATP, the larger product was initiated with GTP, and is therefore not promoter-specific. From the sequence of the pre-melted DNA, the larger RNA either initiated at position -2 on the expected template strand (Fig 5A), or it initiated at position-3 on the expected non-template strand. The pattern of transcription using [α-^32^P]ATP is essentially identical to the overlapped patterns where [γ-^32^P]ATP and [γ-^32^P]GTP were used, indicating that almost all transcription initiated with a purine, and there are few RNAs that initiated with a pyrimidine nucleotide. In the presence of Mtf1, synthesis of the ~45 nucleotide product is almost completely suppressed, and there is considerable suppression of transcripts initiated with GTP. This result clearly demonstrates that a major function of Mtf1 in mitochondrial transcription is to inhibit the non-specific activity of Rpo41.

### Relative strengths of pre-melted and linear promoters

**Fig 6:**
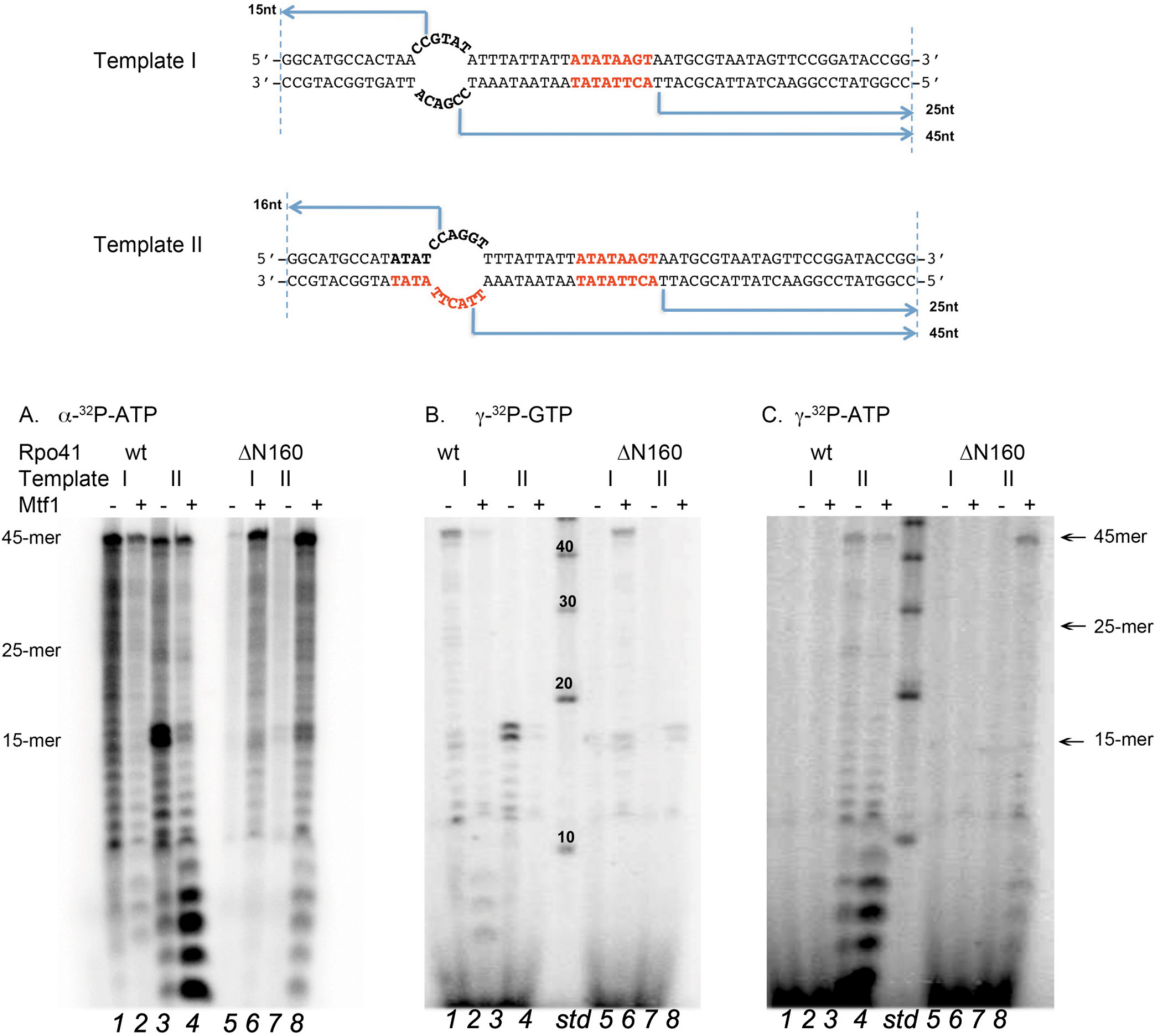
Comparisons of promoter strength and structure. (A). Sequences and structures of the 65-bp constructs contain a linear promoter and a pre-melted region with or without 14S rRNA promoter. (B-D). transcripts yielded from the ab0ove constructs using Rpo41 wild-type or ΔN160 (0.5 μM) in the absence or presence of Mtf1 (2.5 μM), labeled with [γ-^32^P]GTP, [γ-^32^P]ATP, and [α-^32^P]ATP, respectively.

To better understand the template sequence and structural preference for transcription initiation by Rpo41, we designed two oligonucleotide partial duplexes that allow a direct comparison of promoter strength in the pre-melted and linear form (Fig 6). Both constructs (65 bp) contain a 14S rRNA duplex promoter preceded by a pre-melted bubble that is either unrelated to a Rpo41 promoter (Construct I) or contains a 14S rRNA promoter sequence on one strand (Construct II). For clarity, the strand containing the template strand of the duplex promoter is referred to as the T strand, the other is referred to as the NT strand. Each construct thus contains three potential initiation sites: two within the unpaired region, one on each of the T and NT strands, and a 14S rRNA duplex promoter on the T strand. Transcripts from each site can be easily distinguished both by their initiating nucleotide and by the length of the run-off product RNA: a 45-mer from the unpaired region on the T strand, a 15-mer or a 16-mer from the unpaired region on the NT strand, and a 25-mer from the linear promoter on the T strand. Three parallel reactions were performed using [γ-^32^P]ATP, [γ-^32^P]GTP, and [α-^32^P]ATP to aid identification of the initiation sites and the yield of total RNA.

Even in the presence of Mtf1, neither DNA construct directed synthesis of significant amounts of a 25-nucleotide RNA product (Fig 6A, lane 2&4 and 6C lane 2&4). These experiments were conducted at a molar ratio of enzyme:DNA of 1:10, thus there should not be interference between enzyme molecules at closely spaced potential transcription start sites. The apparent affinity of Rpo41 for an open DNA structure (i.e., a pre-melted bubble) is therefore sufficiently high to prevent the enzyme from recognizing a strong promoter sequence in duplex form.

In the absence of Mtf1 and using [α-^32^P]ATP, transcription using construct I yielded a ladder of products > 10 nucleotides in length and with a clearly defined band of 45 nucleotides (Fig 6A lane 1&3). As expected from the sequence of the pre-melted region in construct I, essentially no transcripts initiated with ATP (Fig 6C, lane 1). Labeling the product RNAs with [γ-^32^P]GTP revealed that the reactions products are all G starts: the 45-mer that initiated on the single-stranded region on the T strand, and the bands of 14 and 15 nucleotides that presumably originate from transcription on the single-stranded region on the NT strand (Fig 6B, lane 1, 3). Addition of Mtf1 to the reaction resulted in a major inhibition in the incorporation of [α-^32^P]ATP (Fig 6A, lane 2), in agreement with the experiment shown in Fig 5B(2), where [γ-^32^P]ATP was used to label transcription initiation events. Initiation on the NT strand was completely suppressed and the amount of run-off product on the T strand was reduced by 5-fold. (Table 2) All transcripts on Construct I are initiated at non-promoter sequences and thus the experiments provide additional evidence that an important function for Mtf1 is to suppress non-specific transcription catalyzed by Rpo41.

**Table 2:** Selective inhibitory effect of Mtf1 on holoenzyme activity.

	(-) Mtf1	(+) Mtf1 +	(+)Mtf1/(-)Mtf1
Promoter-specific transcripts 45-mer RNA from T-strand	1.5	1	0.7
Non-specific transcript RNA-mer RNA from the NT-strand	11.9	2.6	0.2
Abortive transcript Accumulated (3–7 mer)	22.7	71.1	3.1
Total transcripts	36.1	74.7	2.1

In the absence of Mtf1, the non-specific single-stranded sequence on the NT strand of Construct II is preferentially used to initiate transcription; surprisingly, it is even preferred over the single-stranded promoter sequence on the T strand (Fig 6B, lane 3). At least in part, this may reflect the specific template sequences, which on the NT strand allows initiation with GTP rather than ATP. Even though all mitochondrial promoters are initiated with ATP, because Rpo41 has evolved from T7 RNAP, the preference of the phage enzyme for initiating with GTP may have been preserved. Mtf1 has a distinct differential effect on the distribution of transcripts on Construct II. Synthesis of the GTP-initiated RNAs on the NT strand is severely inhibited; the amount of the 15/16 long transcripts is reduced at least six-fold, whereas that of the 45-mer from the single-stranded promoter on the T strand is only slightly affected (Fig 6B, lane 4 and Table 2). Thus, by suppressing non-specific activities, Mtf1 makes a positive contribution to selective promoter-directed transcription.

Interestingly, the Rpo41-ΔN160 mutant enzyme has a different response to the presence of Mtf1. In the absence of a transcription factor, Rpo41-ΔN160 catalyzes little synthesis on either Construct I or II (Fig 6A, lane 5&7, 6B, lane 5&7 and 6C, lane 5&7), observations consistent with those described above when using the pre-melted promoter DNA However, in the presence of Mtf1, both specific and non-specific transcriptions are enhanced (Fig 6A, lane 6&8, 6B, lane 6&8 and 6C, lane 6&8). Thus, loss of residue 1–160 in Rpo41 reduces Mtf1’s selective inhibition of non-specific activity.

## Discussion

Despite their identical catalytic role in phosphoryltransfer reactions, RNA polymerases exhibit high structural diversity. Bacteriophages and viruses have the simplest RNAPs that consist of only a single subunit enzyme. Bacterial RNAPs are typically comprised of a 4- or 5-subunit core enzyme that functions by complex with a sigma factor to form a holoenzyme. Eukaryotic transcription is significantly more complex. For example, the core of yeast RNA Pol II is a 12-subunit enzyme that then requires a group of general transcription factors to initiate RNA synthesis. In multi-subunit RNAPs, the activities are divided among the polymerase and its auxiliary proteins. One advantage for recruiting transcription factors is thought to achieve tight regulation over an increased number of promoters. Because of the vast structural differences between single-subunit and multi-subunit RNAPs, it is not immediately clear how the functional division of labor is achieved. Mitochondrial RNA transcriptional system (mtRNAP) is an ideal case for understanding the interplay between the RNA polymerases and transcription factors, as the system contains a catalytic subunit Rpo41 and only single transcription factor Mtf1.

### Rpo41 alone has DNA structural but no sequence specificity

Previously, Rpo41 was previously reported to possess intrinsic promoter-specific transcription activity. However, the experiments used only three NTPs, which precluded detection of non-specific transcripts. Upon a more detailed examination, we found that this activity of Rpo41 is not sequence-specific but is actually template-structure dependent, as Rpo41 has high affinity to pre-melted bubble DNA, and initiates efficiently with either GTP or ATP on a single stranded DNA within the bubble, predominantly from a site that is two nucleotides from the downstream duplex. The activity can be affected by the size of the bubble [18]. Caution should therefore be taken when interpreting Rpo41 transcription reactions on pre-melted templates. Nonetheless, the high non-specific activity could enable mtRNAPs to function as a primase to provide primers for DNA synthesis at the replication fork, as mitochondria lacks a designated primase.

### Rpo41 only possesses promoter specificity in the presence of Mtf1

Because Rpo41 was thought to possess intrinsic promoter specific activity, the function of Mtf1 was assumed to be restricted to promoter binding and unwinding. In this study, while we confirmed that Mtf1 is absolutely required for promoter-specific activity, we also show that Mtf1 increases promoter specific transcription by suppressing Rpo41 non-specific RNA synthesis. On a pre-melted promoter bubble template, Mtf1 biases Rpo41 transcription to that on the template strand by preferentially inhibiting reactions initiated on the NT strand. This could be accomplished by specific interactions between Mtf1 and the NT strand [17, 18], which prevents Rpo41 translocation on the NT strand. The holoenzyme thus confers unidirectionality on the template strand. This mechanism requires holoenzyme to distinguish the T from the NT strand. Given that neither Rpo41 nor Mtf1 exhibits sequence specificity, we propose conformational changes must be induced either by Rpo41-Mtf1 interactions, by the DNA template, or both.

**Fig 7:**
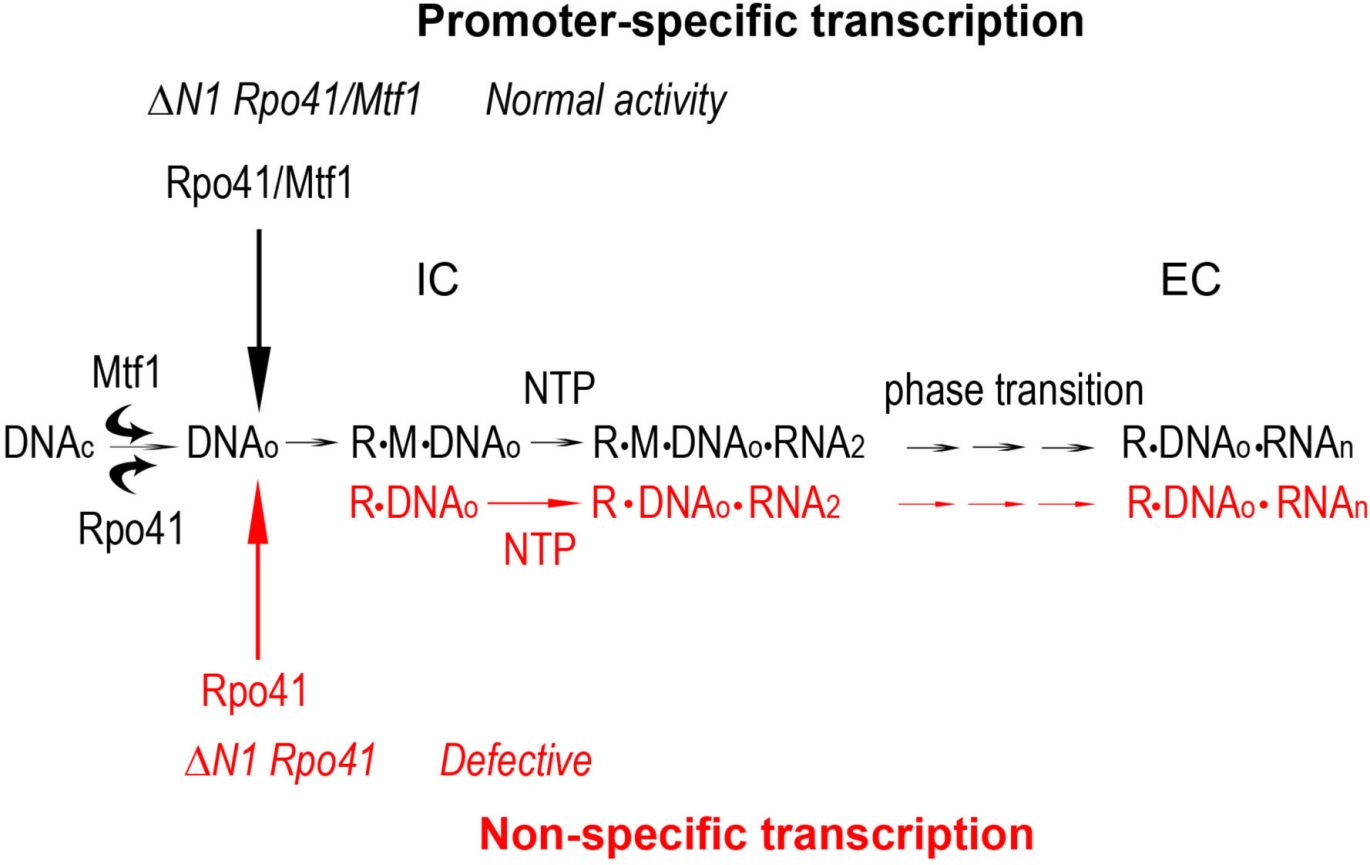
Summary of Rpo41 and Mtf1 roles in RNA transcription. The Rpo41 exhibits promoter-specific activity on closed or open template in the presence of Mtf1 (black pathway). The holoenzyme containing Rpo41 ΔN1 deletion mutant presents normal activity. However, Rpo41 alone exhibits only non-specific activity on both closed and open promoter (red). Rpo41 ΔN1 deletion mutations alone are defective in transcription.

The selective inhibition of non-specific transcription by Mtf1 appears to be intimately related to the N1 subdomain of Rpo41, because holoenzyme with deletion mutant Rpo41-ΔN160 is less sensitive to Mtf1 than wild-type. The N1 subdomain and Mtf1 may jointly interact with the NT strand to inhibit non-specific transcription. Therefore, holoenzyme containing Rpo41-ΔN1 deletion would result in easier promoter release and higher efficiency to transition into elongation phase. The trade-off for N1 deletion is relaxed specificity, as the mutant holoenzymes have elevated both non-specific and specific activities. This, in turn, exemplifies the necessity of Rpo41 N1 and Mtf1 for promoter specific transcription. A summary of our findings is presented in Fig 7.

The defects of the Rpo41-ΔN2 mutant Rpo41 has reduced affinity to both Mtf1 and promoter DNA than wild-type, indicating although this region is spatially homologous to the AT-rich minor groove recognition loop of T7 RNAP, the N2 region forms part of the Mtf1 binding site and performs a distinct function.

Our findings that Mtf1 restores Rpo41-ΔN1 are in good agreement with previous studies [23]. Yeast cells carrying an N-terminal 185 aa deletion mutant of Rpo41 showed normal mRNA levels, but is temperature-sensitive for growth on glycerol media [23]. Cells carrying a more extended deletion of 373 residues, thereby truncating the N2 subdomain, were unable to grow in glycerol, indicating a more severe mitochondrial dysfunction. The mutants also exhibited a reduced mtDNA copy number, providing strong supporting evidence that mtRNAP is an integral part of mtDNA replication.

### Transition from initiation to elongation transcription

To synthesize mature RNAs, all transcription reactions have to transition from initiation and elongation phases, where RNAP releases the promoter and synthesizes RNA processively. Nevertheless, the mechanisms differ: Single subunit RNAP such as T7 RNAP undergoes conformational changes to refold in the NTD promoter-binding site to RNA exit tunnel for processivity enhancement [26, 27, 28, 29], whereas Multiple subunit polymerases such as RNA PolII dispenses transcription factors to open the RNA exit channel [30]. Yeast mitochondrial transcription system contains a single subunit Rpo41 but has a transcription factor Mtf1, and the mechanism for phase transition is unknown. This study indicates that Rpo41 N1 domain and Mtf1 both participate in the process. Mtf1 enhances the number of transcriptional events, but reduces the efficiency of elongation, which is the expense for accurate initiation of transcription at a promoter probably by increasing energy barrier for IC-EC transition. This conclusion agrees with the independent measurements where it was shown that Mtf1 increases the synthesis of abortive transcripts [18] and Rpo41 activity reported in other studies [7, 9]. Whether Rpo41 undergoes drastic conformational changes during phase transition similarly to T7 RNAP awaits for further studies.

## Materials and Methods

### Construction of Rpo41 variants

Rpo41 deletion mutants were constructed where 52, 96, 160, 254, 321 or 393 amino acids from the N-terminus were systematically removed, yielding N-terminal his-tagged variants Rpo41-ΔN52, ΔN96, ΔN160, ΔN254, ΔN321 and ΔN393. pProExHTb-RPO41 DNA was used as template for mutant generation using PCR method, and the unique restriction sites StuI and BsmI were used for in-frame ligation of a mutant construct. Four forward primers, one for each Rpo41 variant were synthesized:

StuI-52aa-rpo41 Fwd 5’-GAAGGCCTACCTCATCTACAACATCAGG,

StuI-96aa-rpo41 Fwd 5’-GAAGGCCTAAATCGCATGCCTCAGCAG,

StuI-160aa-rpo41 Fwd 5’-GAAGGCCTAAAAATGACCCTAATTTCCC,

StuI-254aa-rpo41 Fwd 5’-GAAGGCCTTCGCAACTGCCAAATTCTG,

To construct an Rpo41 variant, the corresponding forward primer is paired with an invariant reverse primer 5’-GCTTTCGAATGCCTTCTTTTCC 3’ in a PCR reaction. The yield PCR product was purified using QIAGEN PCR cleaning kit), and was subject to restriction enzymatic digestion with StuI and BsmI. The gel purified digested PCR fragment was ligated to pProExHTb vector.

### Construction of Mtf1

The clone pTrcHisC-MTF1 coding for gene of a N-terminal His-tagged Mtf1 was subcloned from the parental clone pGEX2TK-MTF1 that contains gene of a GST-Mtf1 fusion protein [7]. The DNA fragment containing MTF1 gene was generated in a PCR reaction using primers: NheI-MTF1 5’-CTAGCTAGCTCTGTTCCAATCCCTG-3’ ad MTF1-HindIII 5’-CCCAAGCTTTCAACCAGAGTGCTCTG-3’. The PCR fragment was digested with NheI and HindIII, gel-purified and subsequently ligated to pTrcHisC vector (Invitrogen Co.)

Clones of all Rpo41 variants and Mtf1 constructs were confirmed by DNA sequencing at the ICMB Core Facilities of The University of Texas at Austin, and expressed in Rosetta cells.

### Purification of His-Rpo41 and His-Mtf1

Expression and purification of His-Rpo41 in *Escherichia coli* was as described previously [9, 31]. All Rpo41 variants are purified using the same method. Cells were inoculated in LB to an OD_600_ = 0.6–0.7, protein expression is induced by addition of 0.6 mM of IPTG and continuing incubation for 4 hours. Cells are lysed in buffer N [50 mM NaH_2_PO_4_ (pH 8.0), 300 mM NaCl, 0.1% tween 20, and 15% glycerol) with 1mM EDTA by addition of lysozyme and soniation. To remove DNA, ammonium Sulfate and Polymin P were added to the cleared cell lysate to a final concentration of 100 mM and 0.1%, respectively, followed by centrifugation. Additional ammonium sulfate was added to the cell lysate to a final concentration of 50% saturation, and proteins are recovered by centrifugation and redissolved in buffer N. The protein was applied to Ni-NTA using batch-binding mode, and ellutated with buffer N+150 mM imidazole. The elutant was applied to a gel filtration column Superdex 200 16/60 (Amersham Biosciences) in buffer N. Fractions containing Rpo41 was pooled and concentrated.

### Substrates design

The 70 bp linear 14S rRNA promoter containing duplex is composed with oligonucleotides: 5’-ccggaattcattaataatttatttattattatataagtaataaataatagttttatataataagaattcc and 5’-ccggaattcttattatataaaactattatttattacttatataataataaataaattattaatgaattcc; The pre-melt 70bp 14S promoter containing bubble template is composed with oligonucleotides 5’-ccggaattcattaataatttatttattattatatgcagcttaaataatagttttatataataagaattcc and 5’-ccggaattcttattatataaaactattatttattacttatataataataaataaattattaatgaattcc;

And the control 70 bp pre-melt bubble without any promoter is composed with oligonucleotides 5’-cggaattcattaataatttatttattattgcagttgtgataaataatagttttatataataagaattcc and 5’-ccggaattcttattatataaaactattatttatgcttgagctaataataaataaattattaatgaattcc.

All oligonucleoitdes are obtained from Integrated DNA Technology Co. The oligonucleotides are gel purified prior to heat-denaturation at 90° followed by gradual cooling to reanneal at 1 degree/min.

### Analytical Gel filtration chromatography

Samples (0.5 mL) was applied to a superdex 13/300 analytical size exclusion column at flowrate of 0.1 mL/min in running buffer 40 mM Tris-HCl, pH 7.9, 150 mM NaCl, 0.1% Tween 20, 15% Glycerol, 1 mM EDTA, 1mM DTT

### Transcription assays

Transcriptions were conducted using 10 μl of reaction buffer (40 mM Tris-HCl, pH 8.0, 20 mM MgCl_2_, 1 mM DTT). The reaction mixtures contained 0.5 μM Rpo41 and 2.5 μM Mtf1 (unless indicated otherwise) with either 1μM for 70 bp linear, pre-melt bubble DNA templates or 0.2 μM plasmid DNA. The reactions were initiated by addition of nucleotides mixtures: all four NTPs to linear or bubble DNAs containing reactions, but only three nucleotide triphosphates ATP, UTP, and GTP (250 uM each) to plasmid DNA to halt the reaction at 107-mer RNA synthesis. Various labeled nucleotide [γ-^32^P]ATP, [γ-^32^P]GTP, or [α-^32^P]ATP (GE Healthcare) was used in reactions as indicated. The reactions mixtures were incubated for 10 min at 20°C, and stopped by the addition of 10 μl of stop-buffer (100 mM EDTA, 90% formamide, 0.02% bromophenol blue, and 0.02% xylene cyanol). Each sample was then boiled at 95°C for 5 min prior to being loaded on an 20% polyacrylamide gel with 7 M urea to resolve RNA products. After electrophoresis, the gel was exposed to a phosphor screen, scanned on Typhoon Trio instrument (GE Healthcare) to quantify RNA products, and quantified using Quantity One (BioRad).

## Supporting Information

S1 Fig Sequence comparison of T7 bacteriophage and mitochondrial RNA polymerases. (DOCX)
