# Supplementary material for: Yeast Mitochondrial Transcription Factor Mtf1 Determines the Precision of Promoter-Directed Initiation of RNA Polymerase Rpo41
Source: PLoS One. 2015 Sep 2;10(9):e0136879. doi: 10.1371/journal.pone.0136879 (PMC4558008; doi:10.1371/journal.pone.0136879)
Supplement: S1 Fig — (DOCX) [file pone.0136879.s001.docx]

# Supporting information

Sequence comparison of T7 bacteriophage and mitochondrial RNA polymerases

**S1. Fig.** Sequence alignment of T7 RNA polymerase, Rpo41 and human mitochondrial

RNA polymerase (hmRNAP) illustrates that Rpo41 shares the conserved catalytic core

(residues 660-1306 ) with T7 RNAP, but has a N-terminal extension, N1 domain

(residues 1-312) and lower homologous N2 domain (313-659).
